# Effectiveness of an Intervention Aimed at Improving Information for Patients with High Cardiovascular Risk: INFORISK Clinical Trial

**DOI:** 10.3390/ijerph18073621

**Published:** 2021-03-31

**Authors:** Carlos Brotons, Irene Moral, Diana Fernández, Mireia Puig, M. Teresa Vilella, Teresa Puig, LLuís Cuixart, Gemma Férriz, Anna M. Pedro, Roger Codinachs, Mónica Rodríguez, Rubén Fuentes, on behalf of INFORISK Study Investigators

**Affiliations:** 1 EAP Sardenya-IIB Sant Pau, 08025 Barcelona, Spain; imoral@eapsardenya.cat (I.M.); dfernandez@eapsardenya.cat (D.F.); mpuig@eapsardenya.cat (M.P.); mtvilella@eapsardenya.cat (M.T.V.); rfuentes@eapsardenya.cat (R.F.); 2 Epidemiology Department, Hospital de la Santa Creu i Sant Pau, Universitat Autònoma de Barcelona, CIBER Cardiovascular, IIB Sant Pau, 08041 Barcelona, Spain; tpuig@santpau.cat; 3 EAP Dreta de l’Eixample, 08013 Barcelona, Spain; llcuixart@eapdretaeixample.cat; 4 ABS Sagrada Familia, 08017 Barcelona, Spain; gemma.ferriz@sanitatintegral.org; 5 ABS Gaudí, 08017 Barcelona, Spain; anamaria.pedro@sanitatintegral.org; 6 EAP Vic Sud, 08500 Vic, Spain; rcodinachs@eapvic.org; 7 CAP Pare Claret, 08037 Barcelona, Spain; mrodriguezb.bcn.ics@gencat.cat

**Keywords:** primary care, early intervention, educational, heart disease risk factors

## Abstract

Background: The concept of global cardiovascular risk is not usually well understood by patients in consultation. Methods: This was a multicenter, prospective, randomized, open clinical trial of one-year duration to evaluate the effectiveness in reducing global cardiovascular risk with an intervention aimed at high-risk patients to improve information on the cardiovascular risk compared to the usual care. The intervention was focused on providing information about cardiovascular risk in a more understandable way, explaining the best practices to reduce cardiovascular risk, and tailoring information to the individual. Results: Four-hundred and sixty-four subjects participated in the study; 59.3% were men, and the mean age was 61.0 (SD 8.0) years. Significant reductions in systolic blood pressure (SBP) (−3.12 mmHg), body mass index (BMI) (−0.34 kg/m^2^), abdominal circumference (−1.24 cm), and REGICOR cardiovascular risk (−0.63) were observed in the intervention group. Overall, no differences in cardiovascular risk score were observed between groups at the end of follow-up. Conclusions: Providing an easy-to-understand assessment of the cardiovascular risk motivated high-risk patients to adopt a healthier lifestyle and improved cardiovascular risk after one year in the intervention group. Clinicians should assess a patient’s baseline understanding of their CV risk using tools other than absolute risk before making treatment recommendations.

## 1. Introduction

National and international clinical guidelines on cardiovascular prevention [1,2,3] mostly use cardiovascular (CV) risk tables to stratify patients according to their risk and make appropriate clinical decisions. The most widely used risk tables are the equations derived from the SCORE project [4] in Europe and the pooled cohort equation in the United States [5]. In our country, the Framingham equation published in 1998 [6] and recalibrated in the REGICOR study [7] is also used. 

Currently, it is not known whether the use of CV risk in consultations improves CV morbidity and mortality outcomes. The real effect of CV risk information given to patients is also not exactly known [8].

Although there is no clear evidence in this regard, there are studies indicating that patients prefer a graphical presentation of risk rather than a simple communication of risk as a percentage [9]. 

The NICE guidelines [10] also emphasize the importance of communication with the patient so that the true meaning of CV risk is as understandable as possible.

In our setting, the calculation of CV risk is widely implemented in clinical practice, although it is true that there are barriers to its routine use, the most important being the short time in consultation [11]. However, rarely is it explained to patients in a routine, structured, and understandable way what high risk means, its consequences, and how to reduce it. This can have important implications in clinical practice in terms of changes in a patient’s lifestyle, control of risk factors, and adherence to medication.

Furthermore, patients usually do not understand the concept of risk and are usually very tolerant of their own risk situation. Reducing CV risk at the individual level begins with appropriate assessment of individual risk and the effective communication of risk. It is crucial to recognize that patients often do not fully understand clinicians’ methods of expressing risk and risk reduction. There is evidence that individuals find it difficult to comprehend mathematical concepts, and even highly educated people have difficulty with relatively simple numeracy questions [12].

There is a common misperception in which a patient judges his or her risk as lower or higher than predicted [13,14].

Interventions that utilize risk communication and decision aids have not led to sustained changes in CV risk profiles, despite improving patient accuracy of risk assessment [15,16,17].

European guidelines for CV disease prevention [2] advocate that high-risk patients automatically qualify for intensive risk factor evaluation and management. This includes the adoption of a healthier diet, an increase in physical activity, and a prompt intervention on all risk factors. 

We hypothesize that providing patients with CV risk information improves risk perception, control of risk factors, and ultimately, global CV risk. The objective of the present study was to evaluate the effectiveness of an intervention to improve the risk information provided to high-risk patients in reducing global CV risk.

## 2. Materials and Methods

This was a multicenter, prospective, randomized, open clinical trial of one-year duration to evaluate the effectiveness in reducing global CV risk with an intervention aimed at high-risk patients to improve information on the CV risk compared to the usual care.

Patients of both sexes who provided informed consent, lacked a history of previous CV disease, and were between 40 and 69 years old were included. In order to participate, they had to meet at least one of the following criteria: an estimation of CV risk according to SCORE ≥5% or according to REGICOR ≥10% or a relative risk >4 according to SCORE; either diabetes mellitus with proteinuria or with at least one major risk factor (smoking, hypercholesterolemia, or hypertension) or chronic kidney disease with a glomerular filtration rate <30 mL/min/1.73 m^2^ or subclinical atherosclerosis diagnosed by invasive or noninvasive techniques (coronary angiography, nuclear imaging, stress cardio resonance, stress echocardiography, ultrasound carotid plaque) such as aortic aneurysm, carotid atheroma plaque (or intima media thickness ≥1.5 mm), severe calcification of the coronary arteries (coronary calcium >300 U Agaston) or ankle braquial index <0.9).

Patients who suffered from a previous CV disease (coronary heart disease or stroke), any condition that limited their life expectancy to less than one year, mental illness that limited their capacity for self-care, or a medical history of alcohol or drug abuse or who were concurrently participating in another clinical trial were excluded.

This research was approved by the Ethics Committee of IDIAP Jordi Gol. It is included in the international registry of clinical trials with the registration number ISRCTN (ISRCTN18383298) and can be consulted via the web at https://doi.org/10.1186/ISRCTN18383298, accessed on 30 March 2021.

Main outcome variable: global cardiovascular risk according to the REGICOR tool.

Secondary variables: weight, height, smoking, alcohol consumption, physical activity survey (International Physical Activity Questionnaire Short Form) [18], Mediterranean diet survey, systolic and diastolic blood pressure, total cholesterol, LDL cholesterol (LDL-c), HDL cholesterol (HDL-c), triglycerides, glycemia, glycosylated hemoglobin, number of visits made by family doctors and nurses, emergency care visits, and hospital admissions.

Six primary health care centers took part in the study, covering a population of approximately 150,000 inhabitants; five out of six were urban, and one was semi-urban.

Intervention: The intervention was carried out by research nurses working in general practice who were trained specifically in communicating with patients about assessing CV risk, changing lifestyle, and improving healthy habits. The individuals randomly assigned to the intervention group received an intervention focused on the provision of comprehensive information about the meaning of CV risk in a more understandable way in terms of absolute risk, relative risk, and vascular age through the use of figures. This information also explained the best way to reduce their CV risk and was tailored to each individual. For this, iconographic material extracted from the QRISK of the National Health Service of the United Kingdom [19] was used. This visual communication tool, which is very understandable regardless of the education level of the individual, is based on a chart that contains 100 faces representing people with the same risk factors as the evaluated individual. Each face is colored yellow (smiling) or purple (not smiling), indicating the proportion of individuals likely to suffer a cardiovascular event. Patients in the intervention group were visited every three months for one year to monitor the risk and reinforce the measures that could best reduce their CV risk.

Control group patients were visited at the beginning of the study and one year after the baseline visit. 

Sample size calculation: Accepting an alpha error of 0.05 and a beta error of 0.2 in a bilateral contrast, 246 subjects were required in the first group and 246 in the second group to detect a difference equal to or greater than 0.8 units. The common standard deviation was assumed to be 3. A loss to follow-up rate of 10% was estimated.

The subjects were randomly assigned to each group (intervention or control) through an electronic system. Randomization was done in a centralized and stratified manner for each of the participating health centers.

Statistical analysis:

A specific database for the study was developed through a platform developed for the study by the Epidemiology Department of Hospital de la Santa Creu i Sant Pau. 

Data were analyzed according to the randomized group. The analysis was based on global intention to treat (ITT) and stratified by study group.

A general descriptive analysis of the variables included in the study was carried out using absolute and relative frequencies for the qualitative variables, as well as the measures of central tendency and dispersion (mean, standard deviation) for the quantitative variables, stratified by study group. To compare the study groups, the chi-square test was used in the contingency table analysis, and Student’s *t*-test was used for independent data in the analysis of quantitative variables.

To compare the REGICOR scores between the two groups and assess its evolution in each group after one year of follow-up, as well as that of other variables of interest (other modifiable CV risk factors), multilevel repeated-measures mixed-effects models were used that take into account intra- and inter-individual variability in the analyses.

In all cases, the hypothesis tests used were bilateral with a significance level of 0.05. All analyses were performed with the STATA/MP 14.0 statistical package.

## 3. Results

Four-hundred and sixty-four subjects participated in the study, and 49.1% were randomized to the intervention group. Of the entire population, 59.3% were men, and the mean age (SD) of participants was 61.0 years (8.0 years). 

### 3.1. Description of Baseline Characteristics 

Table 1 shows the baseline characteristics of the study population. No significant differences in these characteristics were observed between the two groups.

The mean (SD) total cholesterol was 205.36 mg/dL (41.85 mg/dL), LDL-c was 120.61 mg/dL (37.36 mg/dL), and blood glucose of 130.38 mg/dL (53.05 mg/dL). Additionally, 35% performed low physical activity, and 89.44% had moderate or low adherence to the Mediterranean diet.

### 3.2. End of Follow-Up Analysis

Information was recorded at the final study visit in 87.72% of the participants.

In Table 2, we can observe the differences between the baseline and final visits of the control and intervention groups, as well as the intergroup comparison at the end of the follow-up.

Overall, no differences were observed between groups at the end of follow-up. A significant reduction in systolic blood pressure (SBP) of 3.9 mmHg (*p* = 0.003) was observed in the intervention group, while the decrease was 1 mmHg (*p* = 0.328) in the control group. Regarding diastolic blood pressure (DBP), a significant decrease was observed in the two study groups, with an average reduction of around 2 mmHg (*p* = 0.021 and *p* = 0.023).

The patients in the intervention group also showed a statistically significant reduction in the body mass index (BMI), with a mean reduction of 0.5 kg/m^2^, as well as in the abdominal circumference (mean reduction of 1.6 cm), observing a reduction in the percentage of obese patients from 52% at the baseline visit to 45.9% at the final visit (*p* = 0.033). A reduction in BMI was also observed in the control group, but it was not statistically significant.

At the initial visit, the mean total cholesterol value was 205.4 (SD 41.3). Total cholesterol decreased by an average of 6.5 mg/mL between the initial and final visits. The two groups reduced their total cholesterol, but this reduction was only significant in the control group (from 206.9 mg/dL to 197.38 mg/dL, *p* < 0.001).

No significant differences were observed in the level of physical activity (Table 3). Adherence to the Mediterranean diet improved in the intervention group, in which high adherence to the Mediterranean diet increased from 8% to 16% (*p* < 0.001), with no changes observed in the control group (*p* = 0.463). At the end of follow-up, both study groups behaved similarly in relation to adherence to the Mediterranean diet (0.112), the intensity of physical activity (*p* = 0.827), and adherence to treatment (*p* = 0.689).

The intervention did not have an impact on smoking, but it significantly reduced alcohol consumption in the intervention group, decreasing from 59% of subjects who initially drank to 44.6% of subjects at the end of the study (*p* < 0.001).

During the follow-up period, the study participants visited their family doctor an average of 2.76 (SD 2.62) times (*p* = 0.648 between study groups). Further, 10.60% of the subjects in the control group and 9.15% of the subjects in the intervention group were admitted to the emergency room of the primary health care center or the hospital at least once during follow-up (*p* = 0.67). Twelve patients from the control group required hospital admission, 3 of them for CV reasons (1 acute myocardial infarction, 1 unstable angina, and 1 ischemic stroke/TIA), and 6 patients from the intervention group required hospitalization, but none were for cardiovascular reasons (*p* = 0.25).

### 3.3. Impact of the Intervention

**Figure 1:**
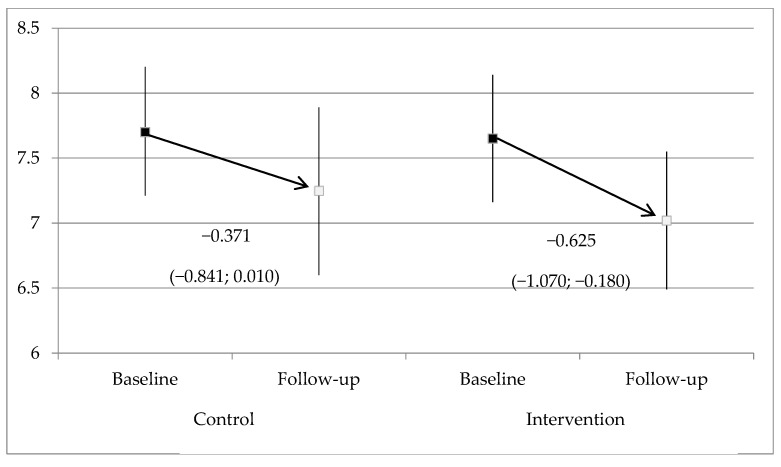
Evolution of REGICOR cardiovascular risk during follow-up by study group (mean and 95% CI).

The REGICOR risk score after the intervention decreased significantly in the intervention group (reduction from 7.65% to 7.02%, *p* = 0.005) but not in the control group (reduction from 7.70% to 7.25%, *p* = 0.059), without registering statistically significant differences between groups in any of the visits (Figure 1).

## 4. Discussion

The results of this clinical trial show that a specific intervention focused on risk communication to patients was effective in improving some CV risk factors in the intervention group (such as SBP, BMI, adherence to the Mediterranean diet, and CV REGICOR risk) after one year. However, no differences were observed between the two groups at the end of the study, presumably due to the fact that some factors were modified in both groups. We also did not find differences between study groups by gender (results not presented). It should be noted that DBP, smoking, and physical activity did not change at the end of follow-up, and surprisingly, total cholesterol decreased in both groups, but it was statistically significant only in the control group. 

Similar to the results of our study, a recently published meta-analysis that examined the effect of lifestyle interventions on CV disease risk factors among workers found that the interventions were effective for SBP (0.66, 95% CI: 0.27–1.60), DBP (0.63, 95% CI: 0.21–1.06), and BMI (0.71, 95% CI: 0.15–1.11) but were ineffective for LDL-cholesterol (0.46, 95% CI: −0.02, 0.93) [20]. A pragmatic randomized controlled trial that assessed the effectiveness of a complex intervention for the primary prevention of CV disease found significant differences (in favor of the intervention group) in DBP and waist circumference [21]. In both studies [20,21], a significant reduction in total cholesterol or LDL cholesterol was not observed in the intervention group.

A systematic review that assessed the effect of providing global coronary heart disease risk information to adults showed that global risk information alone or accompanying education increased the accuracy of perceived risk and increased the intent to start therapy [22].

Studies with repetitive risk information sessions showed small significant reductions in risk prediction (absolute differences of −0.2% to −2% in the next 10 years). Studies that provided information on risk on a single occasion were not successful.

Another systematic review that assessed the effectiveness of methods of communicating probabilistic information to patients found that visual aids and absolute risk formats can improve patients’ understanding of such information, whereas numbers needed to treat can lessen their understanding [23]. 

López-González et al. [24] carried out a study in a population of 2844 young workers who presented a low CV risk. They were randomly allocated to one of three study groups: regular control, measurement of CV risk, or calculation of vascular age. Through this study, a significant reduction in workers’ CV risk in the vascular age group compared to the other two groups was observed. 

Current guidelines for CV disease prevention [1,2,3] highlight the importance of shared decision making and risk communication in CV disease prevention. 

However, these guidelines are not very clear on how to conduct this comprehensive discussion about CV risk with patients. Materials should be developed that allow a better understanding of risk in order to convey the message effectively and achieve greater adherence to treatment. Furthermore, it is important to recognize that while current risk equations perform well for populations, the positive and negative predictive value of these equations can vary greatly. Thus, it is important for clinicians to consider each patient individually.

Before proposing an intervention to reduce risk, clinicians must take time to communicate this risk using the most appropriate and understandable methods, such as figures or relative risk of heart age. 

### Strengths and Limitations

The key strengths of this study included its design, with clear inclusion of high-risk patients purposefully selected to represent this population. It followed one protocol, used standardized methods, and was multicentered in a primary care setting, and nurses were suitably trained for this study. 

The study has some limitations that should be mentioned. The intervention consisted in using different strategies (absolute risk, relative risk, figures, and vascular age). Thus, we cannot know which of the elements included was more important in improving healthy behaviors, risk factors, and CV risk in the intervention group. Another weakness of the study is that the follow-up period was only one year, which could be too short a period of time to evaluate improvements in risk factors and CV risk. Although we observed statistically significant reductions in some clinical parameters, the percentage of reduction at the end of follow-up was moderate and probably does not have a clinical short-term impact at the individual level. However, it potentially could have an important long-term impact, as well as an impact at the population level. 

Our study makes a valuable contribution to our knowledge regarding the effect of an intervention on communication with patients in improving CV risk in high-risk patients. The study showed that changes in lifestyle and CV risk factor led to corresponding changes in CV risks in the intervention group and not in the control group, although we must admit that there were no differences between the two groups at the end of the study, most likely because some positive lifestyle changes were also observed in the control group. 

## 5. Conclusions

Providing an easy-to-understand assessment of the CV risk motivated high-risk patients to adopt a healthier lifestyle and improved CV risk after one year in the intervention group in terms of reducing SBP, BMI, waist circumference, and REGICOR score risk, although no differences in terms of CV risk were found between the two groups at the end of the study. Clinicians should assess a patient’s baseline understanding of their CV risk using tools other than absolute risk before making treatment recommendations. 

## Figures and Tables

**Table 1:** Baseline characteristics of the study participants.

KERRYPNX	Study Group		Total (*n* = 464)	***p*** **-Value**
	Control (*n* = 236)	Intervention (*n* = 228)		
Education level, *n* (%)				0.775
No studies	4 (1.69%)	3 (1.32%)	7 (1.51%)	
Primary	63 (26.69%)	68 (29.82%)	131 (28.23%)	
Secondary	91 (38.56%)	81 (35.53%)	172 (37.07%)	
University/higher degree	77 (32.63%)	76 (33.33%)	153 (32.97%)	
Employment status, *n* (%)				0.902
Self-employed	28 (11.86%)	22 (9.65%)	50 (10.78%)	
Employed worker	69 (29.24%)	72 (31.58%)	141 (30.39%)	
Unemployed	18 (7.63%)	14 (6.14%)	32 (6.90%)	
Retired	99 (41.95%)	97 (42.54%)	196 (42.24%)	
Others	20 (8.47%)	22 (9.65%)	42 (9.05%)	
Hypertension, *n* (%)	173 (73.31%)	161 (70.61%)	334 (71.98%)	0.374
Antihypertensive treatment, *n* (%)	157 (66.53%)	147 (64.47%)	304 (65.52%)	0.642
Dyslipidemia, *n* (%)	171 (72.46%)	175 (76.75%)	346 (74.57%)	0.112
Lipid-lowering treatment, *n* (%)	139 (58.90%)	138 (60.53%)	277 (59.70%)	0.721
Type 2 diabetes, *n* (%)	160 (67.80%)	160 (70.18%)	320 (68.97%)	0.242
Hypoglycemic agents, *n* (%)	146 (61.86%)	145 (63.60%)	291 (62.72%)	0.700
Antiplatelet, *n* (%)	43 (18.22%)	29 (12.72%)	72 (15.52%)	0.102

Percentages and *p*-value were calculated taking into account the category of missing values (values not shown).

**Table 2:** Intragroup and intergroup comparison of risk factors and anthropometric parameters.

Mean (SD) [*n*]	Group				Inter-Group *p*-Value
	Control		Intervention		
	Baseline	End of Follow-Up	Baseline	End of Follow-Up	
SBP (mmHg)	138.08 (15.23) [*n* = 235]	136.80 (14.99) [*n* = 185]	138.16 (15.14) [*n* = 228]	135.04 (14.42) [*n* = 192]	0.139
Intra-group *p*-value	0.328		0.003		
DBP (mmHg)	81.49 (10.42) [*n* = 235]	79.64 (8.99) [*n* = 182]	81.79 (10.23) [*n* = 228]	80.06 (9.12) [*n* = 191]	0.721
Intra-group *p*-value	0.021		0.023		
BMI (kg/m^2^)	31.05 (5.32) [*n* = 233]	31.09 (5.92) [*n* = 164]	31.06 (5.99) [*n* = 227]	30.72 (5.80) [*n* = 183]	0.361
Intra-group *p*-value	0.364		0.047		
Abdominal circumference (cm)	107.29 (13.39) [*n* = 233]	106.68 (13.50) [*n* = 167]	107.13 (13.62) [*n* = 227]	105.89 (13.61) [*n* = 178]	0.317
Intra-group *p*-value	0.381		0.001		
Total cholesterol (mg/dL)	206.88 (43.20) [*n* = 234]	197.38 (44.23) [*n* = 201]	203.77 (40.41) [*n* = 223]	199.75 (41.83) [*n* = 204]	0.356
Intra-group *p*-value	<0.001		0.190		
HDL-c (mg/dL)	55.31 (19.52) [*n* = 231]	53.29 (17.91) [*n* = 199]	53.27 (15.63) [*n* = 215]	50.69 (14.44) [*n* = 203]	0.192
Intra-group *p*-value	0.018		0.024		
LDL-c (mg/dL)	121.48 (39.30) [*n* = 229]	115.37 (37.42) [*n* = 194]	119.67 (35.23) [*n* = 213]	118.79 (35.58) [*n* = 195]	0.300
Intra-group *p*-value	0.010		0.748		
Triglycerides (mg/dL)	181.43 (111.67) [*n* = 232]	175.55 (149.35) [*n* = 195]	188.88 (139.06) [*n* = 219]	164.83 (118.30) [*n* = 197]	0.454
Intra-group *p*-value	0.930		0.094		
Blood glucose (mg/dL)	134.05 (56.58) [*n* = 234]	130.85 (55.17) [*n* = 194]	128.96 (46.32) [*n* = 222]	128.38 (43.14) [*n* = 191]	0.641
Intra-group *p*-value	0.388		0.845		

SD = standard deviation; SBP = systolic blood pressure; DBP = diastolic blood pressure; BMI = body mass index; HDL-c = high density lipoprotein cholesterol; LDL-c = low density lipoprotein cholesterol.

**Table 3:** Evaluation of study groups’ adherence to the Mediterranean diet and to treatment, degree of physical activity, smoking habits, and alcohol intake during the study (intra) and at the end of follow-up (inter).

[*N*] *n* (%)	Study Group				Inter-Group *p*-Value
	Control		Intervention		
	Baseline	Follow-Up	Baseline	Follow-Up	
Adherence to the Mediterranean diet Low Moderate High	[*N* = 235] 47 (20.00%) 160 (68.09%) 28 (11.91%)	[*N* = 170] 24 (14.12%) 128 (75.29%) 18 (10.59%)	[*N* = 227] 47 (20.70%) 161 (70.93%) 19 (8.37%)	[*N* = 181] 21 (11.60%) 131 (72.38%) 29 (16.02%)	0.115
Intra-group *p*-value	0.463		<0.001		
Degree of physical activity Low Moderate Vigorous	[*N* = 235] 84 (35.74%) 125 (53.19%) 26 (11.06%)	[*N* = 170] 66 (38.82%) 84 (49.41%) 20 (11.76%)	[*N* = 227] 80 (35.24%) 116 (51.10%) 31 (13.66%)	[*N* = 181] 74 (40.88%) 84 (46.41%) 23 (12.71%)	0.827
Intra-group *p*-value	0.636		0.151		
Adherence to treatment High Moderate-high Moderate-low Low-moderate Low	[*N* = 226] 143 (63.27%) 70 (30.97%) 8 (3.54%) 4 (1.77%) 1 (0.44%)	[*N* = 156] 126 (80.77%) 21 (13.46%) 4 (2.56%) 1 (0.64%) 4 (2.56%)	[*N* = 218] 129 (59.17%) 68 (31.19%) 17 (7.80%) 3 (1.38%) 1 (0.46%)	[*N* = 163] 133 (81.60%) 24 (14.72%) 5 (3.07%) 0 (0%) 1 (0.61%)	0.689
Intra-group *p*-value	0.002		<0.001		
Smoking habit, *n* (%) Non-smoker Former smoker (<1 year) Former smoker (≥1 year) Smoker	93 (39.41%) 5 (2.12%) 64 (27.12%) 74 (31.36%)	78 (42.16%) 3 (1.62%) 48 (25.95%) 56 (30.27%)	63 (27.63%) 8 (3.52%) 77 (33.92%) 79 (34.80%)	55 (28.80%) 5 (2.62%) 71 (37.17%) 60 (31.41%)	0.609
Intra-group *p*-value	0.206		0.131		
Alcohol intake, *n* (%)	120 (50.85%)	78 (45.88%)	134 (59.03%)	82 (44.57%)	0.979
Intra-group *p*-value	0.052		<0.001		

## Data Availability

The data presented in this study are available on request from the corresponding author. The data are not publicly available due to privacy restrictions.
